# Eave ribbons treated with transfluthrin can protect both users and non-users against malaria vectors

**DOI:** 10.1186/s12936-019-2958-9

**Published:** 2019-09-18

**Authors:** Emmanuel P. Mwanga, Arnold S. Mmbando, Paul C. Mrosso, Caleb Stica, Salum A. Mapua, Marceline F. Finda, Khamis Kifungo, Andrew Kafwenji, April C. Monroe, Sheila B. Ogoma, Halfan S. Ngowo, Fredros O. Okumu

**Affiliations:** 1 Environmental Health and Ecological Sciences Department, Ifakara Health Institute, Morogoro, Tanzania; 2 Institute of Biodiversity, Animal Health and Comparative Medicine, University of Glasgow, Glasgow, G12 8QQ UK; 3 School of Public Health, University of Witwatersrand, Johannesburg, South Africa; 4 Johns Hopkins Center for Communication Programs, Baltimore, MD USA; 5 University of Basel, Basel, Switzerland; 6 Swiss Tropical and Public Health Institute (Swiss TPH), Basel, Switzerland

**Keywords:** Eave ribbons, Ifakara Health Institute, *Anopheles arabiensis*, Malaria, Transfluthrin, Tanzania, Outdoor biting, Spatial repellents, Mosquito traps, Push–pull

## Abstract

**Background:**

Eave ribbons treated with spatial repellents effectively prevent human exposure to outdoor-biting and indoor-biting malaria mosquitoes, and could constitute a scalable and low-cost supplement to current interventions, such as insecticide-treated nets (ITNs). This study measured protection afforded by transfluthrin-treated eave ribbons to users (personal and communal protection) and non-users (only communal protection), and whether introducing mosquito traps as additional intervention influenced these benefits.

**Methods:**

Five experimental huts were constructed inside a 110 m long, screened tunnel, in which 1000 *Anopheles arabiensis* were released nightly. Eave ribbons treated with 0.25 g/m^2^ transfluthrin were fitted to 0, 1, 2, 3, 4 or 5 huts, achieving 0, 20, 40, 60, 80 and 100% coverage, respectively. Volunteers sat near each hut and collected mosquitoes attempting to bite them from 6 to 10 p.m. (outdoor-biting), then went indoors to sleep under untreated bed nets, beside which CDC-light traps collected mosquitoes from 10 p.m. to 6 a.m. (indoor-biting). Caged mosquitoes kept inside the huts were monitored for 24 h-mortality. Separately, eave ribbons, UV–LED mosquito traps (Mosclean) or both the ribbons and traps were fitted, each time leaving the central hut unfitted to represent non-user households and assess communal protection. Biting risk was measured concurrently in all huts, before and after introducing interventions.

**Results:**

Transfluthrin-treated eave ribbons provided 83% and 62% protection indoors and outdoors respectively to users, plus 57% and 48% protection indoors and outdoors to the non-user. Protection for users remained constant, but protection for non-users increased with eave ribbons coverage, peaking once 80% of huts were fitted. Mortality of mosquitoes caged inside huts with eave ribbons was 100%. The UV–LED traps increased indoor exposure to users and non-users, but marginally reduced outdoor-biting. Combining the traps and eave ribbons did not improve user protection relative to eave ribbons alone.

**Conclusion:**

Transfluthrin-treated eave ribbons protect both users and non-users against malaria mosquitoes indoors and outdoors. The mosquito-killing property of transfluthrin can magnify the communal benefits by limiting unwanted diversion to non-users, but should be validated in field trials against pyrethroid-resistant vectors. Benefits of the UV–LED traps as an intervention alone or alongside eave ribbons were however undetectable in this study. These findings extend the evidence that transfluthrin-treated eave ribbons could complement ITNs.

## Background

Vector control is the frontline malaria prevention strategy across Africa today. Insecticide-treated nets (ITNs) and house spraying with residual insecticides (IRS) alone contributed 81% of all gains accrued against malaria between 2000 and 2015 [1]. In Tanzania, effective vector control interventions contributed to a 50% reduction in malaria prevalence between 2008 and 2017 [2, 3], and the country is now pursuing an ambitious new strategy to reduce malaria prevalence below 1% by 2020 [4]. Today, ITNs and IRS continue to be the main interventions deployed against malaria vectors, however gaps in protection can remain due to various challenges, notably insecticide resistance [5], sub-optimal ITN access and use [6], high net attrition rates [7], and high levels of exposure to malaria vectors occurring outdoors or indoors before bed time [8, 9].

To complement these current strategies and advance efforts towards malaria elimination, complementary new tools are urgently required. In recent years, substantial effort has been put into creating an expanded vector control toolbox [10]. Some of these are techniques widely used in historical times with great success and are being brought back to the fore, such as larval source management [11] and mosquito-proof housing [12]. Others are the commodity-based approaches, such as insecticide-treated hammocks [13], insecticide-treated clothing [14], topical repellents [15], insecticides propagated by mosquitoes, i.e. auto dissemination [16], eave tubes combined with house screening [17], insecticide-treated livestock [18], entomopathogenic fungi [19], attractive targeted sugar baits [20], odour-baited mosquito traps [21], targeted spraying of mosquito swarms [22] and spatial repellent based technologies [23, 24]. Unfortunately, none of these so far have proven as scalable as ITNs in low-income communities. Moreover, many still require high user compliance, which particularly hinders interventions such as topical repellents [25].

Spatial repellents are increasingly being proposed as a leading contender in the complementary vector control toolbox, especially since they can protect multiple persons at the same time and often function at sub-lethal doses [23]. Another advantage is that recent advances have demonstrated new delivery formats that require no electricity, remain effective over several months and have multiple modes of action against mosquitoes, including bite prevention, feeding inhibition and lethality [26–29]. In one example, hessian-based strips treated with transfluthrin prevented at least 75% of outdoor-biting by *Anopheles arabiensis*, *Culex* and *Mansonia* mosquitoes for at least 1 year [29]. Combinations of these spatial repellent products and odour-baited mosquito traps have also been evaluated, against indoor and outdoor-biting mosquitoes [30–33].

Mmbando et al. [32] recently described a related application, i.e. eave ribbons, which are simple hessian ribbons treated with spatial repellents and wrapped around eaves of houses (without completely blocking the eave-spaces) to prevent outdoor-biting and indoor-biting mosquitoes over long periods of time. In the initial studies, they demonstrated high levels of protection in semi-field settings against laboratory-reared mosquitoes and also in experimental hut studies against field mosquitoes in rural Tanzania. It was concluded that this technology, pending further development and validation, has potential to complement current tools, as it is simple, low-cost, highly-scalable and easy-to-use; making it suitable even for poorly-constructed houses and low-income groups [31].

In a follow up study [31], Mmbando et al. evaluated different configurations of a push–pull system consisting of the transfluthrin-treated eave ribbons and odour-baited traps. Here, they concluded that efficacy of push–pull was mainly due to the spatial repellent component, and that adding odour-baited traps only slightly improved personal protection, but excessive trap densities increased exposure outdoors [31].

This current study addressed two important questions that remained unanswered regarding the eave ribbon technology. First, whether the eave ribbons at different intervention coverages, could effectively provide both personal and communal protection without diverting mosquitoes from users to non-users, as previously shown with transfluthrin-based mosquito coils under incomplete coverage situations [34]. Second, whether the personal and communal protection could be improved through different configurations of the push–pull systems, such as using indoor traps instead of outdoor traps as previously used [31], or placing the ribbons and traps in different houses instead of the same house as previously tested [30, 31, 35, 36]. For purposes of this study, a commercially-available UV–LED trap recently demonstrated as effective for trapping Tanzanian *Anopheles* and *Culex* mosquitoes was used as the intervention trap of choice alongside the eave ribbons [37].

## Methods

### Mosquito tunnel and experimental huts

**Fig. 1:**
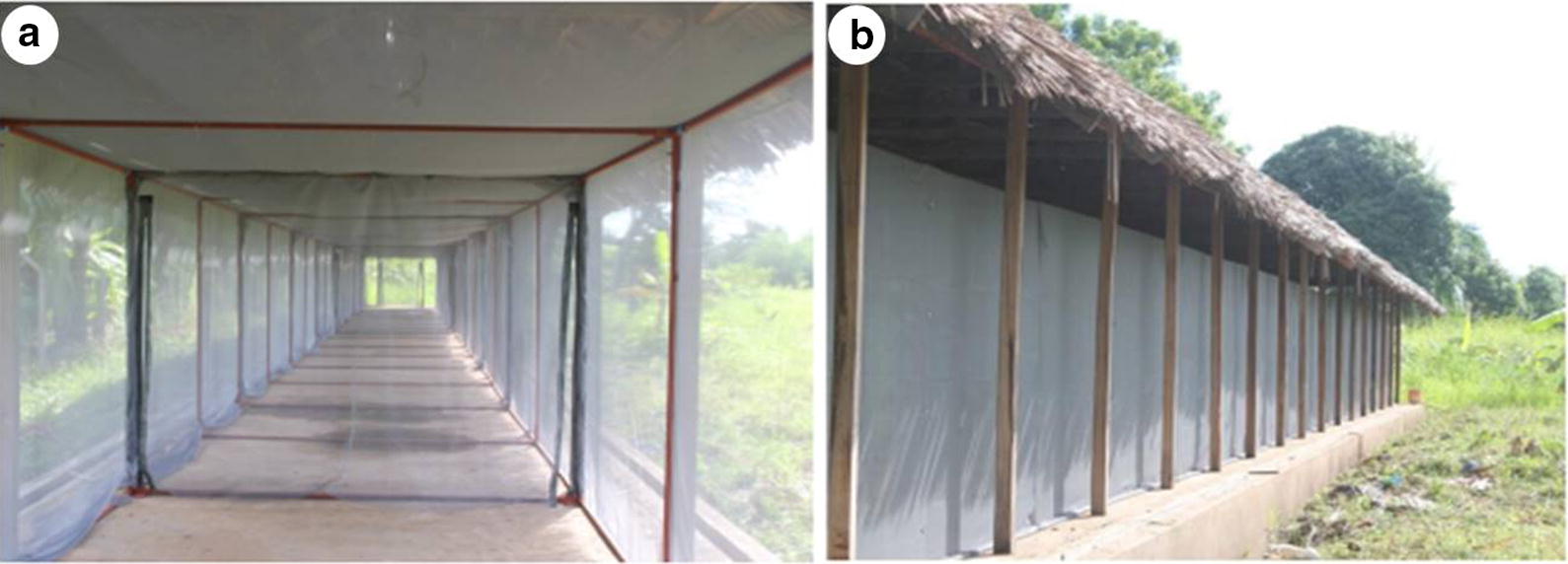
Pictorial illustration showing the internal view of the long, screened tunnel when empty (**a**), and an outer view of the long screened tunnel (**b**)

This study was conducted inside a long, screened tunnel at Ifakara Health Institute’s Mosquito City Facility in Tanzania (Fig. 1). This system is 110 m long, 3 m wide and 2.5 m high, and was designed to allow free movement of adult mosquitoes while interacting with hosts, houses or interventions. Often referred to as the Mosquito Marathon Tunnel, this system has previously been used for evaluating effects of distance and direction on mosquito responses to interventions such as attractants [38], odour-baited devices [39] or push–pull [32], as well as effects of new vector control technologies on densities and survival of mosquitoes [39, 40].

Five portable experimental huts (3.0 m length × 2.7 m width × 2.0 m height) were constructed and positioned inside the tunnel, 20 meters away from each other. Four huts were regularly fitted with either transfluthrin treated eave ribbons or UV–LED mosquito traps as described below while one hut that neither had eave ribbons nor UV–LED mosquito traps acted as the control and was also used to measure effect to non-users.

### Eave ribbons and mosquito traps

The eave ribbons were made with hessian fabric treated with 0.25 g/m^2^ of transfluthrin as previously described by Mmbando et al. [31, 32]. This technology was originally designed for low-income households with gaps on eaves and walls. They are wrapped around open eaves without completely blocking the spaces, and are meant to protect from both indoor-biting and outdoor-biting mosquitoes. Previous versions of the eave ribbons were treated with higher doses of transfluthrin, and resulted in > 99% bite prevention indoors and outdoors. The 0.25 g/m^2^ dose had initially provided 77% protection leaving enough protection gap for improvements. It was therefore selected for subsequent studies that evaluated the combination of the ribbons and traps in push–pull settings [31, 32]. Once treated, hessian materials retain efficacy for 6 months years [29].

**Fig. 2:**
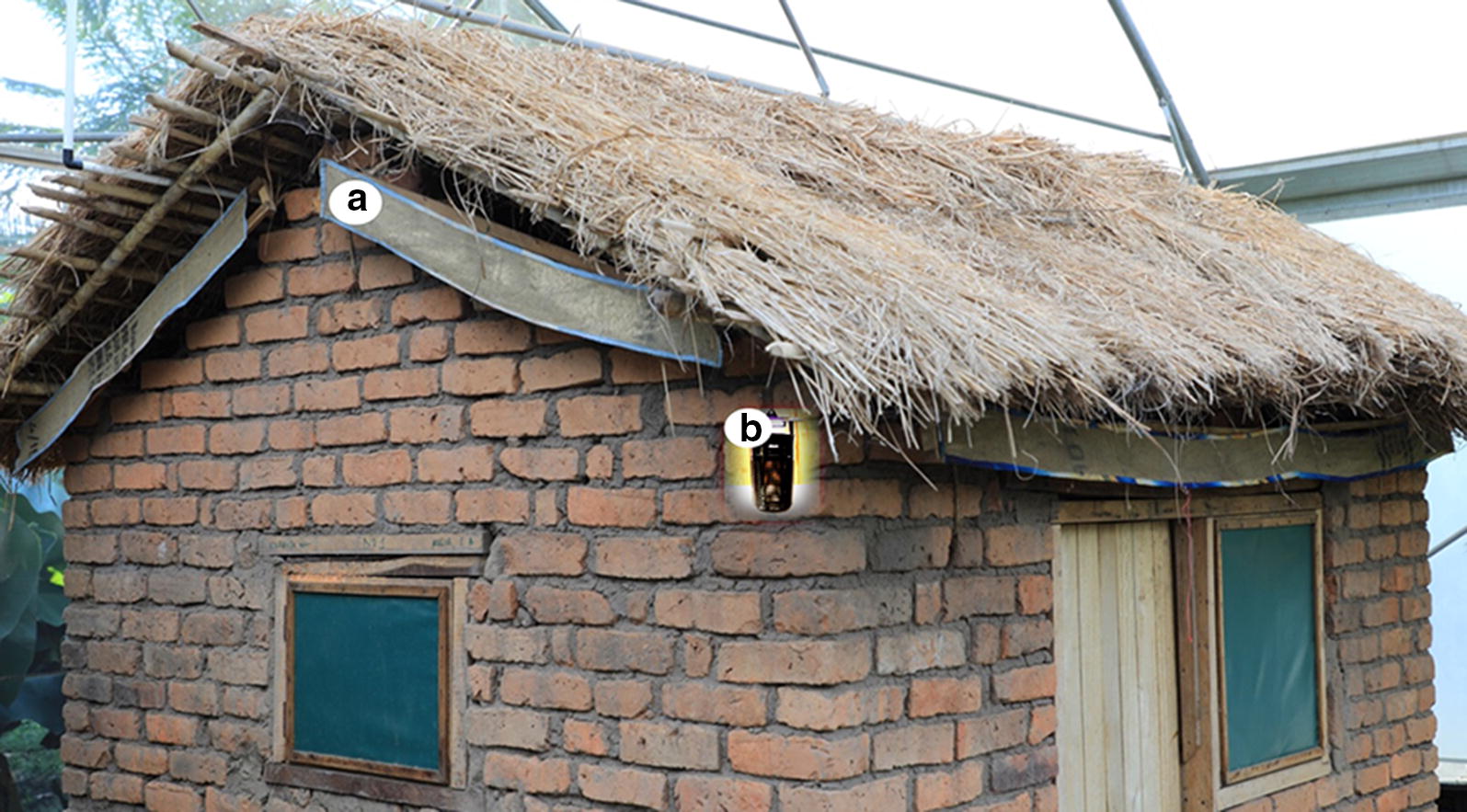
Illustration of transfluthrin-treated eave ribbons wrapped around the eave space (**a**), and Mosclean trap (**b**). In this study, the Mosclean trap was used as an intervention and was placed always indoors. A separate indoor sampling trap, CDC-light trap was used for measuring efficacy of intervention. The eave ribbons and traps were assessed individually, or in push–pull system by putting them in the same houses or adjacent houses

The selected trap was a new LED trap (Mosclean), which attracts mosquitoes by emitting 365 nm ultraviolet (UV) LED light and generates CO_2_ gas by photocatalytic reaction (Seoul-Viosys, Korea). The trap has recently been described in detail [37], and caught at least as many mosquitoes as human volunteers, or other previously used traps in semi-field and field tests [37]. Unlike in Mmbando et al. study which used odour-baited traps placed outdoors [31, 32], the Mosclean trap, used in this current study was placed indoors. Both eave ribbons and traps are illustrated in Fig. 2.

### Mosquito releases and recaptures

Laboratory-reared female *Anopheles arabiensis* were used, all obtained from colonies maintained at the Mosquito City facility. Each night, 1000 hungry adult females (3–5-days old) starved for 6 h by removing sugar meals from their cages, were released in the tunnel in batches of 250 between every two huts to ensure equal distribution throughout the tunnel.

Consenting adult male volunteers assisted in assessing biting risk outdoors and indoors as follows: one volunteer sat outdoors near each of the five huts and performed human landing catches (HLC) from 6 p.m. to 10 p.m. each night to trap mosquitoes attempting to bite them. The volunteers then moved inside the huts, slept under untreated bed nets and turned on a CDC-light trap placed next to the bed to catch host-seeking mosquitoes from 10 p.m. to 6 a.m. the next day. This procedure was first described in detail by Mmbando et al. [32], and enabled evaluation of both outdoor-biting risk (HLC catches) and indoor-biting risk (CDC-light trap catches) in ways generally representative of typical human-mosquito interactions in and around east African households [8, 9]. These studies showed that most people are outdoors between 6 and 10 p.m. doing different household chores, before going indoors and eventually sleeping from 10 p.m. to 6 a.m. Though there were variations in actual times that people went indoors, we fixed the mosquito collection hours to the approximate the observed averages.

CDC-light traps and HLC were selected for being an effective and practical method for catching *Anopheles* mosquito indoors and outdoors respectively. All huts were thoroughly cleaned and left unused for 2 days between different experiments or upon change of intervention, to minimize transfluthrin residual effects.

### Assessing protective efficacy afforded to users and non-users at different percentage coverages of transfluthrin-treated eave ribbons

A baseline assessment of biting risk indoors and outdoors was first conducted for five nights with all the five huts having no intervention. Thereafter, protective efficacy was assessed at 20%, 40%, 60%, 80% and 100% coverage with transfluthrin-treated eave ribbons, each time for five consecutive nights. To assess efficacy at 20% coverage one of the five huts was randomly selected and fitted with the transfluthrin-treated eave ribbons, the remaining four huts being non-users. Next, two of the five huts were randomly selected and fitted with transfluthrin-treated eave ribbons, to achieve 40% coverage. Similarly, three, four or five huts were fitted with the treated eave ribbons to achieve 60%, 80% and 100% coverage respectively, all randomly assigned. Assessment of the protective efficacy in all five huts was done as described above. Based on estimates from previous experiments, these tests were conducted for a maximum of 30 nights, thus achieve satisfactory statistical power. Five nights were regarded as control (zero coverage) followed by 25 nights of treatment, five for each level of coverage. Each morning, all mosquitoes not recaptured were removed using electric aspirators.

### Assessing protective efficacy of transfluthrin-treated eave ribbons alone, Mosclean UV–LED traps alone, or combinations of the two interventions (i.e. push–pull) against indoor-biting and outdoor-biting risk among users and non-users

**Fig. 3:**
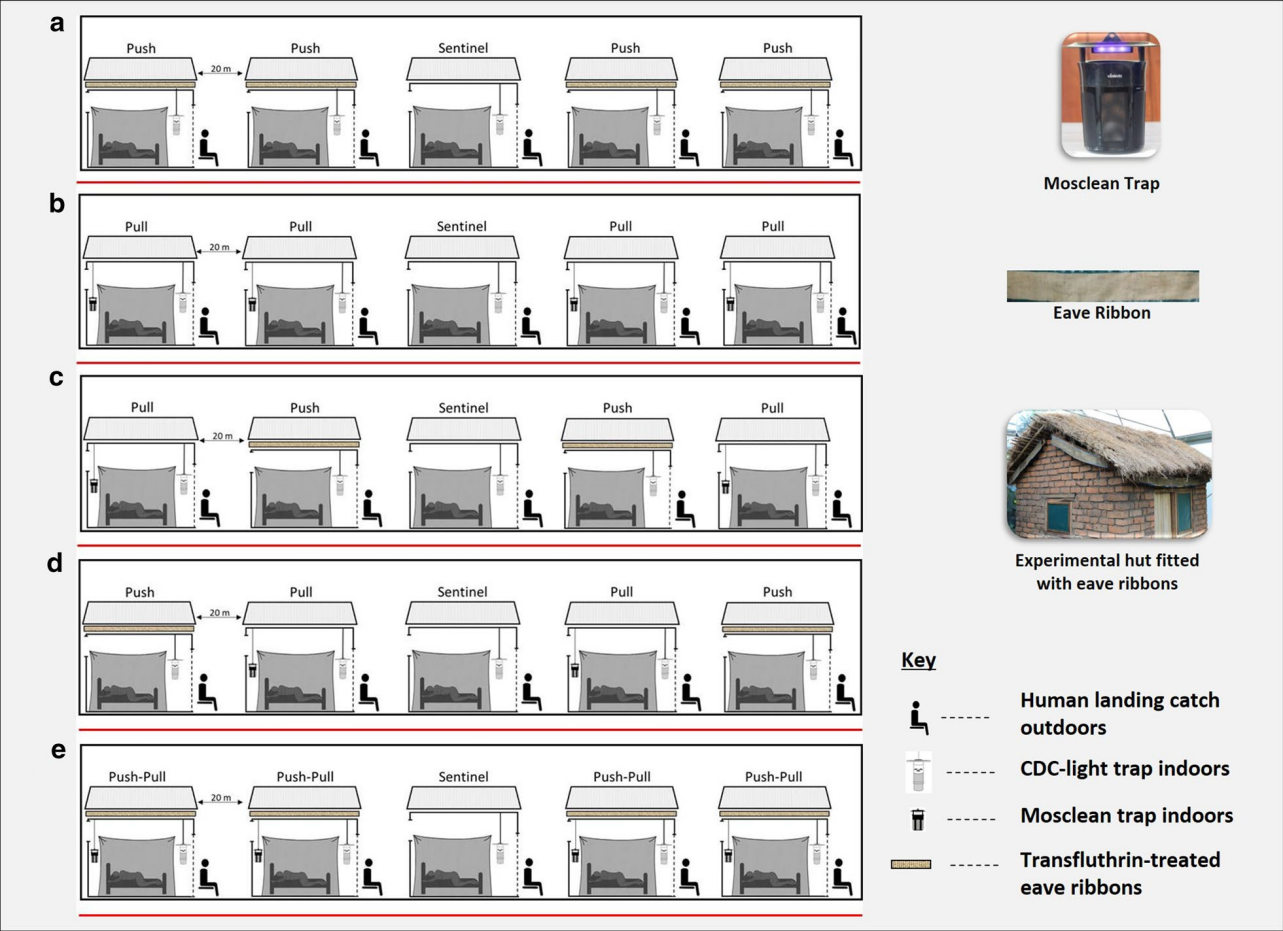
Illustration of the experimental setup to evaluate, protective efficacy of: push component only, i.e. all huts except non-user have transfluthrin-treated eave ribbons (**a**), and pull component only, i.e. all huts except non-user have Mosclean traps (**b**), push–pull mosaic, where the transfluthrin-treated eave ribbons, i.e. push, and Mosclean traps, i.e. pull are in different huts (**c**, **d**), and standard push–pull system, where both eave ribbons and traps are in the same hut (**e**). Each night, adult consenting volunteers caught mosquitoes attempting to bite them outdoors from 6 to 10 p.m., then moved indoors under bed nets, beside which CDC-light traps caught mosquitoes from 10 to 6 a.m.

The two different interventions were first evaluated individually in two separate experiments. All huts except the central non-user hut (also referred to us the sentinel hut) were fitted with either transfluthrin-treated eave ribbons alone (Fig. 3a), or Mosclean traps alone (Fig. 3b). Outdoor biting risk (from 6 to 10 p.m.) and indoor biting risk (from 10 p.m. to 6 a.m.) was measured as described above using HLC and CDC-light traps respectively. For each intervention, five nights pre-intervention period of mosquito collections was included to establish natural biting risk profile indoors and outdoors.

The third experiment was designed to assess potential of combining the push component (eave ribbons) and pull component (Mosclean traps) in mosaic format, where the two were not fitted to same hut as previously tested [31], but rather in separate huts. All huts except the sentinel hut were fitted with either transfluthrin-treated eave ribbons alone (push) or the Mosclean trap alone (pull) as described in Fig. 3c, d. The sentinel hut always remained without either of the interventions and represented both a natural control and a non-user household. This push–pull mosaic experiment was done twice, first, when huts with eave ribbons were nearest to the non-user hut (Fig. 3c), and second when huts with Mosclean traps were nearest to the non-user hut (Fig. 3d). The experiments were each conducted for 15 consecutive nights, the first five nights being controls and the subsequent nights being intervention period.

Lastly, the efficacy of standard push–pull design, where all huts except sentinel are fitted with both traps and transfluthrin-treated eave ribbons was evaluated (Fig. 3e). Assessment of efficacy was conducted similar to the push–pull mosaic for 15 nights.

### Assessing mortality of mosquitoes exposed inside huts with or without transfluthrin-treated eave ribbons

In this experiment, all huts except the sentinel hut were fitted with transfluthrin-treated eave ribbons. Netting cages, each containing 50 female *An. arabiensis* mosquitoes were suspended inside each of the five huts, 1.5 metres above ground, and left for 12 h overnight. The mosquitoes were then transferred to a separate room (average temp: 27 ± 2 °C; relative humidity: 80 ± 5%) and maintained on 10% glucose solution, then monitored for 24-h mortality. This experiment was also conducted for 15 nights. The first five nights were control nights, during which caged mosquitoes were introduced, but none of the huts had eave ribbons. This was followed by 10 nights during which mosquitoes were placed inside same huts, but this time four of the huts were fitted with transfluthrin-treated eave ribbons.

### Data analysis

Data analysis was done using R statistical software, version 3.5.2 [41]. Comparisons of nightly collections were made between individual huts (treated and untreated) and also between periods before and periods after introducing the interventions. Number of mosquitoes collected indoors and outdoors, before and after treatment was compared by fitting Generalized Linear Mixed Models (GLMMs), using *lme4* package [42]. Mean mosquito catches was first averaged for all the five huts for each of the five night pre-intervention periods to ascertain the baseline exposure levels indoors and outdoors.

To assess impact of interventions, mosquito counts were modelled following negative binomial distributions, with the main effect being treatment, i.e. whether huts had eave ribbons, traps or both, depending on experiment. Experimental nights and hut ID were included as random effects to account for variations between nights and huts. Protective efficacies, i.e. percentage protection accruable from the different interventions at the different huts, were calculated using model estimated means from pre-intervention and intervention nights using the formula, 100 * (Control–Treatment)/Control.

Any percentage protection observed in huts without the interventions was considered to represent community level protection affordable to non-users, while protection in houses with interventions was considered a combination of personal and community-level protection for users from interventions in their own huts and interventions in the neighboring huts. Graphical representations of the findings were created using R graphics package *ggplot2* [43].

## Results

### Protective efficacy of transfluthrin-treated eave ribbons afforded to users and non-users at different intervention coverages

**Fig. 4:**
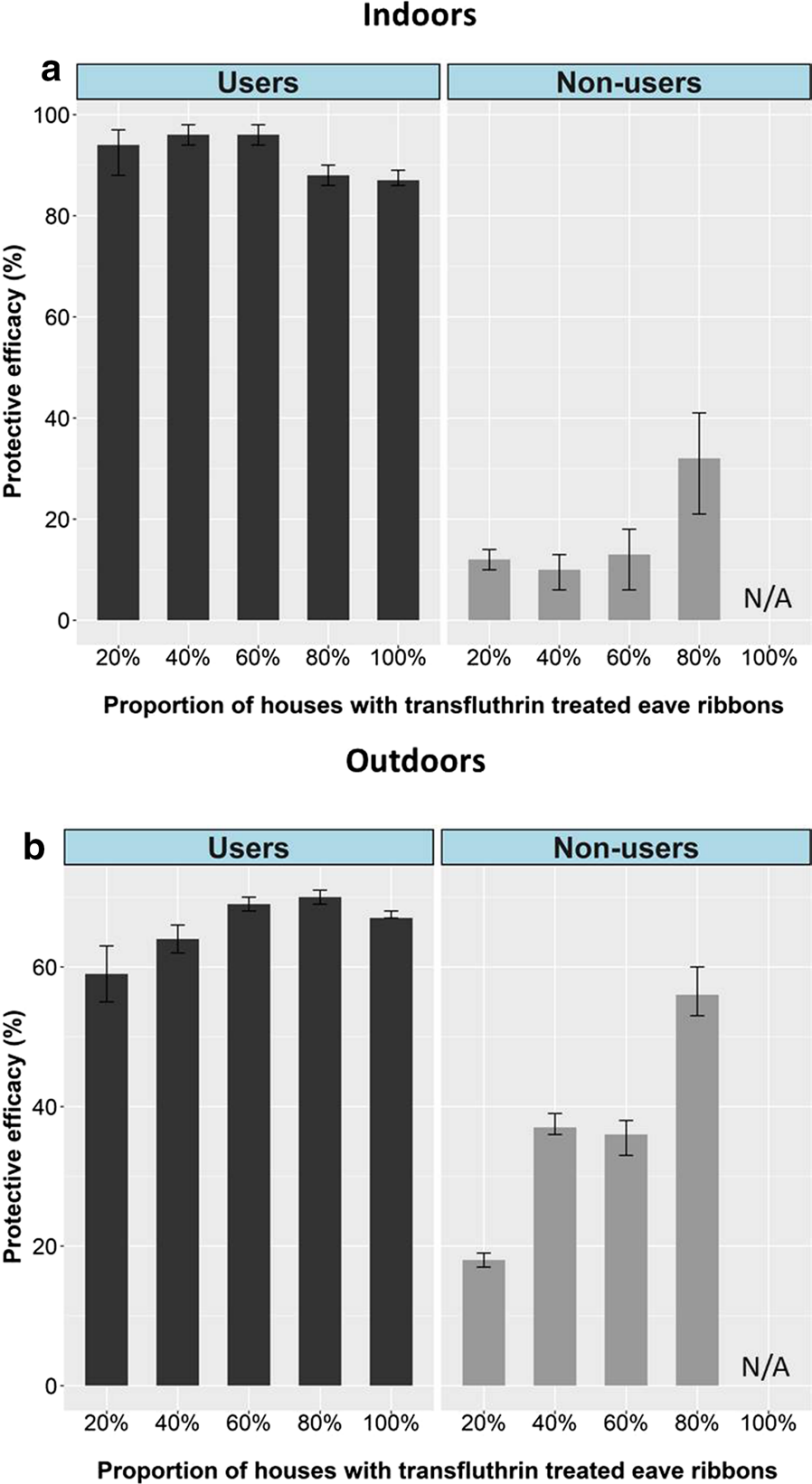
Protective efficacy of transfluthrin treated eave ribbons with increase in coverage for users and non-users, indoors (**a**), outdoors (**b**). Coverage levels were as follows: 0% (none of the huts was fitted with eave ribbons), 20% (one hut fitted with eave ribbons, the other four being non-users), 40% (two huts fitted with eave ribbons, the other three being non-users), 60% (three huts fitted with eave ribbons, the other two being non-users), 80% (four huts fitted with eave ribbons, the remaining one being non-user), and 100% coverage (all five huts fitted with eave ribbons)

A summary of the findings is provided in Fig. 4. In comparison to control nights before introducing the eave ribbons, the number of mosquitoes caught indoors was reduced by more than 80% in all huts fitted with transfluthrin-treated eave ribbons. This percentage protection remained constant for users even when overall coverage increased from one of five huts (20%) to five of five huts (100%). Protection for users against outdoor exposures was 59% at 20% coverage, but slightly improved as coverage increased to a maximum of 69 protection at 60% coverage. Outdoor protection remained above 60% between 40% and 100% eave ribbon coverage.

For non-users, protection against indoor mosquito bites was low (< 20%) between 20 and 60% coverage levels, with modest 32% protection once eave ribbons coverage reached 80%. Outdoor protection for non-users was modest (between 20 and 60% protection) but it increased to as high as 56% protection once intervention coverage reached 80%.

### Protective efficacy of transfluthrin-treated eave ribbons alone, Mosclean traps alone, or combinations of the two interventions (i.e. push–pull) against indoor and outdoor-biting risk among users and non-users

**Fig. 5:**
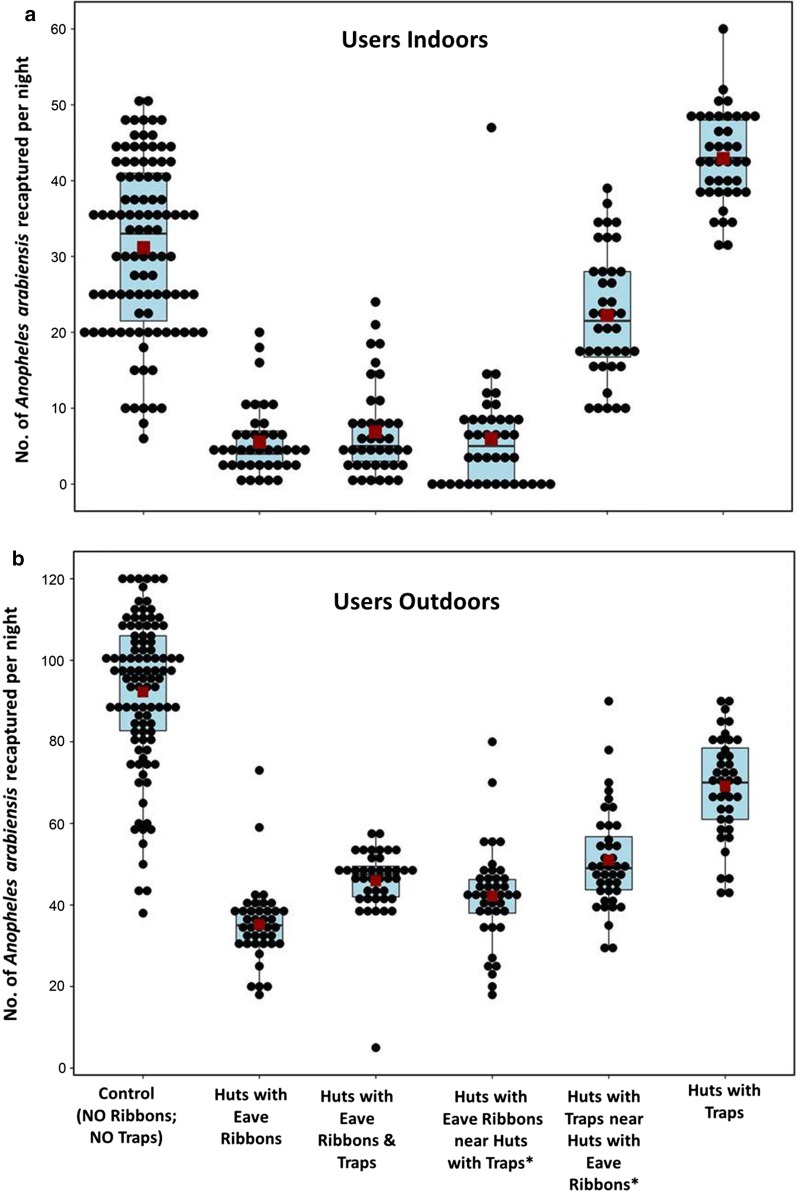
Number of *Anopheles arabiensis* females recaptured per night indoors (**a**) and outdoors (**b**) at different huts of intervention users. Both median (black line crossing the box plot) and estimated means (red square inside the boxplot) are shown. Each black dot represents actual number of mosquitoes recaptured in different experimental nights. All the huts had intact untreated bed nets for basic protection. Observations were made before fitting traps or transfluthrin-treated eave ribbons (i.e. controls), and after the huts were fitted with either the eave ribbons (huts with eave ribbons), traps (huts with traps) or both eave ribbons and traps fitted to same hut (i.e. push pull). There were also observations, based on push–pull mosaic system, where half of the user huts had eave ribbons and the other half had traps (*)

**Fig. 6:**
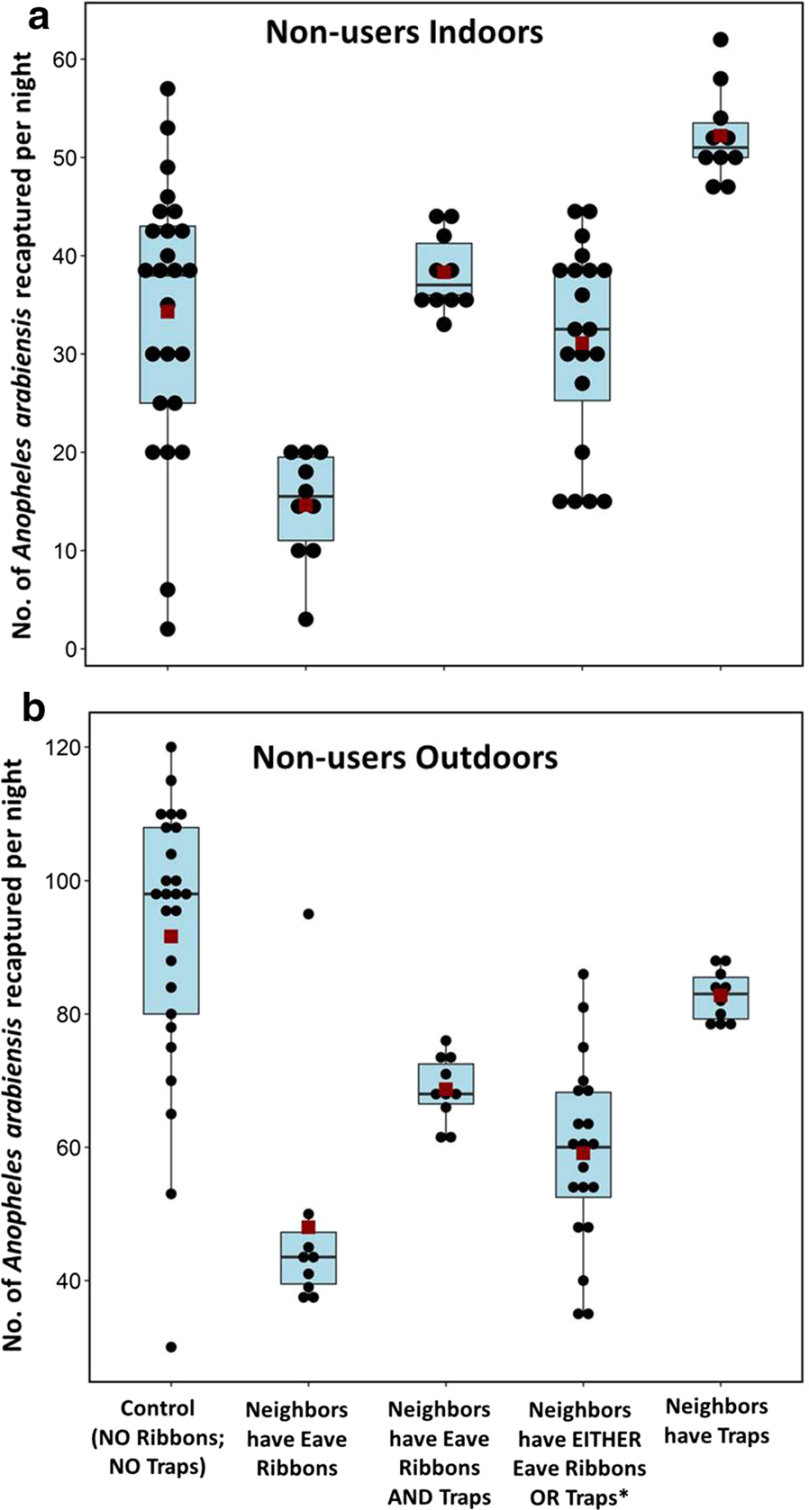
Number of *Anopheles arabiensis* females recaptured per night indoors (**a**) and outdoors (**b**) at the hut of the non-user (i.e. the sentinel hut). Both median (black line crossing the box plot) and estimated means (red square inside the boxplot) are shown. Each black dot represents actual number of mosquitoes recaptured in different experimental nights. All the huts had intact untreated bed nets for basic protection. Observations were made at the sentinel non-user hut before fitting the neighboring huts with traps or transfluthrin-treated eave ribbons (i.e. controls), and then after the neighbors were fitted with either eave ribbons, traps or both traps and eave ribbons fitted to same hut. There was also observations based on push–pull mosaic system, where half of the neighbors huts had eave ribbons, and the other half had traps, i.e. Neighbors have EITHER traps OR eave ribbons (*)

Results of the different experimental designs including mean nightly catches in houses with and without the different interventions, and their associated protective efficacies are summarized in Table 1 and Figs. 5, 6. In the experiment where all huts except the sentinel hut were fitted with transfluthrin-treated eave ribbons, mosquito biting in the user huts was reduced by 83% indoors (Mean of 5.4 bites/night compared to 31.2 bites/night before intervention), and 62% outdoors (35.2 bites/night compared to 92.2 bites/night before intervention). Protection in the non-user hut was 57% indoors (14.6 bites/night compared to 34 bites/night before the intervention) and 48% outdoors (48 bites/night compared to 92 bites/night before the intervention).

**Table 1:** Mean nightly recaptures of malaria mosquitoes in huts with and without intervention

Intervention	Mean nightly recaptures in huts with intervention/users (data from intervention huts combined)					Mean nightly recaptures in huts without intervention/non-users (data from sentinel hut only)				
	Total no. test nights	Indoors		Outdoors		Total No. test nights	Indoors		Outdoors	
		Mean recaptures ± 2SE (∑)	% Reduction (p values)	Mean recaptures ± 2SE (∑)	% Reduction (p values)		Mean recaptures ± 2SE (∑)	% Reduction (p values)	Mean recaptures ± 2SE (∑)	% Reduction (p values)
Control (all huts have only bed nets)	100	31.2 ± 2.2 (3118)	–	92.2 ± 3.7 (9222)	–	25	34.3 ± 5.3 (857)	–	91.6 ± 8.2 (2291)	–
Traps only (all huts except sentinel have Mosclean traps beside the bed nets)	40	43.3 ± 1.9 (1717)	− 38.1 (p < 0.001)	69.1 ± 3.9 (2765)	25 (p < 0.001)	10	52.2 ± 2.9 (522)	− 52.3 (p < 0.001)	82.8 ± 2.3 (828)	9.6 (p > 0.05)
Spatial repellents only (all huts except sentinel have transfluthrin-treated eave ribbons)	40	5.4 ± 1.4 (221)	82.6 (p < 0.001)	35.2 ± 3 (1409)	61.8 (p < 0.001)	10	14.6 ± 3.4 (146)	57.4 (p < 0.001)	48 ± 10.6 (480)	47.6 (p < 0.001)
Push–pull mosaic (all huts except sentinel are fitted with either Mosclean trap or transfluthrin-treated eave ribbon)	40 *(in huts with ribbons)*	5.7 ± 2.5 (239)	81.9 (p < 0.001)	42.2 ± 3.7 (1688)	54.3 (p < 0.001)	20	31.1 ± 4.5 (622)	9.3 (p > 0.05)	59.1 ± 6.1 (1182)	35.5 (p < 0.001)
	40 *(in huts with traps)*	22.6 ± 2.6 (889)	27.8 (p < 0.001)	51.0 ± 3.8 (2040)	44.7 (p < 0.001)					
Push–pull (all huts except sentinel are fitted with both traps and transfluthrin-treated eave ribbons)	40	6.5 ± 1.9 (275)	79.1 (p < 0.001)	46 ± 2.6 (1836)	50.2 (p < 0.001)	10	38.3 ± 2.4 (383)	− 11.7 (p > 0.05)	68.7 ± 3.1 (687)	25 (p < 0.001)

Standard errors (95 confidence interval), and the percentage reduction in biting risk are also shown

Outdoor biting was assessed by human landing catches, while indoor biting was assessed by CDC-light traps in all huts, including those that had Mosclean traps as the intervention

In the experiment where all huts except the sentinel hut were fitted with Mosclean traps, mosquito densities indoors, as measured by CDC light traps increased by 38%, but there was modest biting reduction of 25% outdoors. The same effect was observed in the non-user hut (the sentinel hut), where indoor densities increased by 52%, and outdoor biting decreased by 10% (Table 1 and Fig. 6).

In the push–pull mosaic experiment, where eave ribbons and traps were used but in different huts, the huts with transfluthrin-treated eave ribbons received protection of 82% and 54% indoors and outdoors respectively. Huts with Mosclean trap received 28% and 45% protection indoors and outdoors respectively. The hut without intervention received only 9% protection indoors and 36% protection outdoors (Table 1 and Fig. 6). Figures 5 and 6 also demonstrate that when the huts with Mosclean traps were near huts with transfluthrin-treated eave ribbons, the risk of mosquito bites was substantially lower than when all the intervention huts had Mosclean traps.

Lastly, in experiments evaluating the standard push–pull design, where the trap and spatial repellent were used in the same hut, users received 79% protection indoors and 50% protection outdoors. This approach however did not provide any protection to non-users indoors, but rather slightly increased exposure by 12%. There was however modest protection to the non-user outdoors by 25%.

### Number of mosquitoes caught by Mosclean traps

**Fig. 7:**
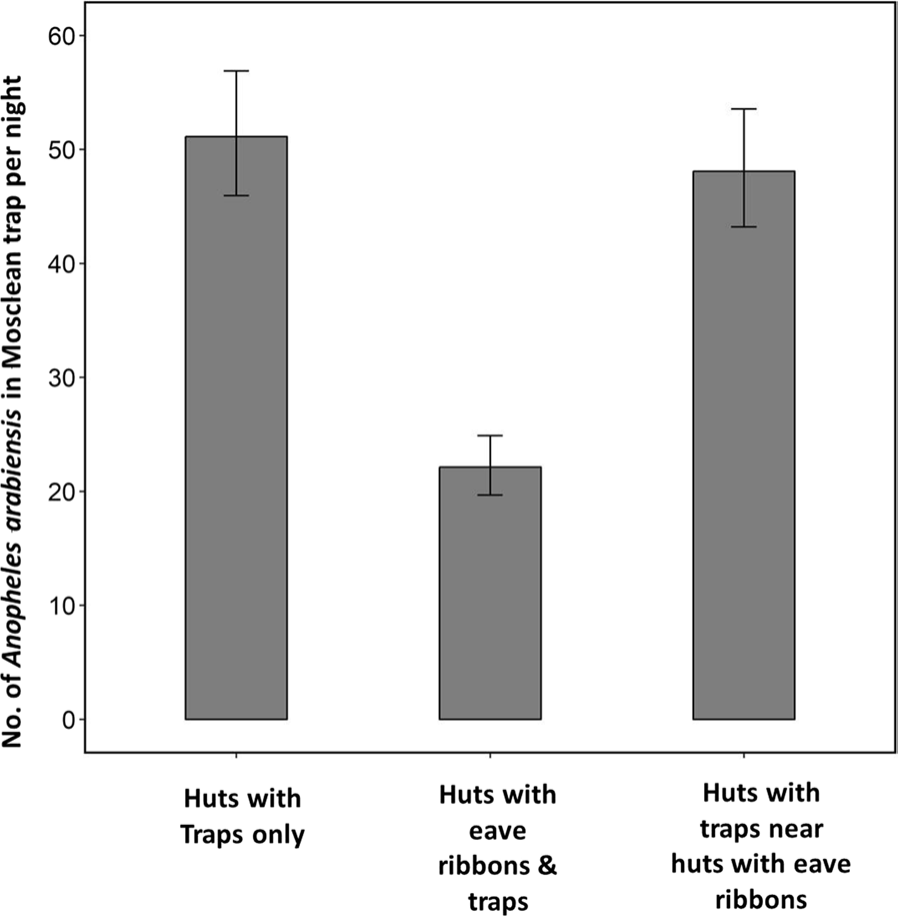
Mean number of *Anopheles arabiensis* mosquitoes recaptured by CDC-light traps inside huts fitted with the UV–LED trap (Mosclean) alone or Mosclean trap and transfluthrin-treated eave ribbons. Catches in huts with Mosclean Trap near huts with eave ribbons are also shown. The error bars represent the 95% confidence intervals

The number of mosquitoes caught by Mosclean traps varied between the three different settings where they were used (Fig. 7). When Mosclean was used alone in the huts, it caught an average (and 95% confidence interval) of 51.1 (46.0–56.9) mosquitoes per night. When used inside huts adjacent to other huts with eave ribbons, the catches were similar, i.e. 48.1 (43.2–53.5) per night. However, when this trap was used inside huts fitted with the transfluthrin-treated eave ribbons, the mean mosquito catches were reduced by half to just 22.1 (19.7–24.9) mosquitoes per night.

### Mortality of mosquitoes exposed inside huts with or without transfluthrin-treated eave ribbons

**Fig. 8:**
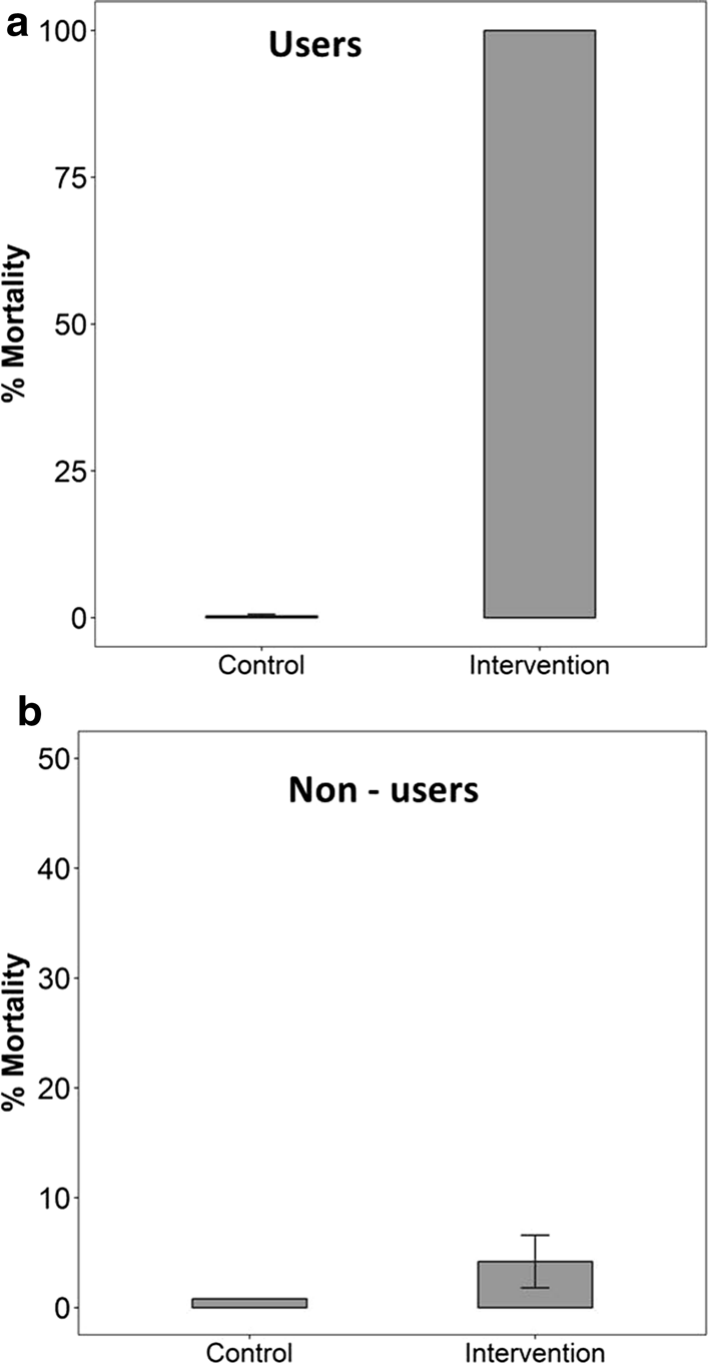
Percentage mortality of mosquitoes exposed in huts with or without transfluthrin-treated eave ribbons, i.e. user huts (**a**) and non-user huts (**b**). The error bars represent the 95% confidence intervals

The mortality rates in user huts and non-user huts are shown in Fig. 8, for the periods before introducing the intervention (i.e. control), and the period during which the transfluthrin-treated eave ribbons were in place (i.e. intervention). Average mortality of 100% was observed in mosquitoes held overnight inside huts with transfluthrin-treated eave ribbons, compared to 4% mortality observed in the non-user hut. Mortality before introducing the ribbons was negligible in all huts.

## Discussion

This study assessed whether transfluthrin-treated eave ribbons, previously demonstrated to protect against both indoor-biting and outdoor-biting mosquitoes in semi-field and experimental hut studies [31, 32], could effectively protect both users and non-users without diverting mosquitoes from users to non-users. Another question addressed was whether the protection offered by these ribbons, either at personal or communal level could be improved by adding mosquito traps to form push–pull, and by different configurations of such push–pull systems. Two main differences in the configurations were as follows: (a) this current study used indoor intervention traps instead of outdoor traps as previously used [31], and (b) this study included one configuration with spatial repellents and traps in different houses and a another with both interventions placed in the same house as previously tested [30–33, 36]. Overall, the studies lasted more than 450 hut nights, during which mosquito trapping was done indoors and outdoors, totalling more than 900 trap nights.

The most important finding was that transfluthrin-treated eave ribbons can protect both users and non-users. Besides, the protection afforded to the non-users peaked when the intervention was delivered at high coverages. In the tests where four of the five huts had the intervention, and the sentinel hut (i.e. non-user) did not, the eave ribbons provided 83% and 62% protection indoors and outdoors respectively to users, plus 57% and 48% protection indoors and outdoors to the non-user. Separately, in the experiment where coverage was varied from 0 to 100%, protection for users remained constant after 20% coverage while protection for non-users increased correspondingly with intervention coverage, peaking once 4/5 (80%) of huts had received the eave ribbons. Protection for users was consistently higher indoors than outdoors, possibly due to: (1) surface area of the transfluthrin treated eave ribbons relative to the indoor space, (2) the preference of candidate vector species, *An. arabiensis* to bite more outdoors than indoors [44], (3) sub-lethal effect of transfluthrin, which may disrupt host-seeking behaviour of mosquitoes entering the huts [28], and (4) the barrier effect of eave ribbons, however minimal [32]. The findings regarding personal protection to users corroborate those from the previous studies which tested the efficacy of the transfluthrin treated eave-ribbons at both semi-field and field settings [32, 33]. However, the findings relating to non-users represent the first report of such additional benefits associated with the eave ribbons technology.

An earlier study by Maia et al. [34] suggested that under conditions of incomplete coverage, transfluthrin-based products, in this case mosquito coils, provided personal protection to users, but could potentially divert mosquitoes to other users. In this current study, there was no increase in exposure to the non-users, possibly because the intervention also killed substantial proportions of the mosquitoes exposed in those huts, and which would have otherwise been diverted to the non-users. Consistently, 100% of mosquitoes held inside the huts with transfluthrin-treated eave ribbons, died within 24 h of observation. Such levels of mortality indicate not only diminished likelihood of diversion of mosquitoes, but also suggest increased communal protection. However, it will be important to validate these findings in field settings where malaria mosquitoes are resistant to pyrethroids, and where such mortality may be reduced. Preliminary findings from rural south-eastern Tanzanian villages suggest that transfluthrin vapours remain substantially toxic to *An. arabiensis, Anopheles funestus* and *Culex* mosquito populations, which are known to be resistant against pyrethroids and carbamates (Mmbando et al., unpublished). The first experimental hut study with eave ribbons in rural Tanzania also showed significant repellency against all these mosquito species despite being pyrethroid resistant. Nonetheless, this must be investigated in detail to guide future potential application of the eave ribbons technology.

These findings add to the body of evidence that spatial repellent eave ribbons can provide protection against malaria vectors both indoors and outdoors in the peri-domestic space. The benefits extend beyond personal or household protection, and are accruable also non-users. Studies of night time human activities and sleeping patterns highlight the importance of interventions that can protect people during times when ITN use is not feasible, including when they are outdoors or indoors before going to sleep. Spending time outdoors near the home during evening hours, is common in many parts of sub-Saharan Africa [8], particularly in rural communities [8]. This includes activities such as cooking and other household chores, resting, and socializing in the hours before bed [8, 9, 45]. Spatial repellent eave ribbons offer a potentially viable solution for protecting people during these times and can offer protection to groups of people without requiring high levels of individual compliance.

Mosclean traps (pull component) alone resulted in increased risk of exposure to indoor mosquito bites in both the huts with taps and the sentinel hut without traps. Besides, it offered only minimal protection against mosquito bites outdoors. This trap, which has UV–LED and a photocatalytic CO_2_ generator as the main lures, was recently shown to be highly attractive and therefore effective for trapping mosquitoes indoors [37]. It could have therefore increased the overall densities indoors as measured by the CDC-light trap in the same huts. This also indicates that Mosclean may be attracting large densities of the mosquitoes to the vicinity, but it does not trap all the mosquitoes. In past experiments where CO_2_ baited traps were placed outdoors at the peri-domestic area, the traps provided only marginal protection against *An. arabiensis* indoors, but increased biting risk to volunteers sitting outdoors [31]. An interesting observation however was that when the huts with the traps were near huts with transfluthrin-treated eave ribbons, the risk of mosquito bites was substantially lower than when all the huts had traps (Figs. 5, 6). The protective efficacy of the eave ribbons was, therefore, more obvious than that of the Mosclean traps at the scale of these studies. Mass trapping of mosquitoes generally has cumulative benefits, which could not be detected in this study since new batches of mosquitoes were released nightly.

Using the traps together with eave ribbons, whether in same houses or in different houses achieved less protection for non-users compared to eave ribbons alone (Table 1). For users, the protection achieved was similar to that obtained with just eave ribbons alone. The change from standard push–pull system to push–pull mosaic resulted in modest improvements in protection indoors or outdoors for the non-user, but these gains were still lower than what was achievable by transfluthrin-treated eave ribbons alone (Table 1). Moreover, in the push–pull mosaic design, the specific user huts that had Mosclean traps achieved lower percentage protection both indoors and outdoors than huts fitted with eave ribbons. The motivation for push–pull mosaic, as opposed to standard push–pull where huts are assigned both traps and repellents, had been to minimize any antagonistic effects between the two interventions at hut level, and also reduce costs and complexity of the overall application package. A related observation was that actual mosquito catches by the Mosclean traps themselves were halved upon introduction of eave ribbons, suggesting any value of these devices for removal trapping would be compromised by the spatial repellents.

Considering the complexities associated with using traps, especially those that require baits, Mmbando et al. [31] suggested that once high coverage with transfluthrin-treated eave ribbons is achieved, it may be best to not add traps at all. Nevertheless, since traps remove large numbers of mosquitoes from circulation, it is still theoretically possible that large-scale deployment may deliver communal level benefits overtime, as previously simulated by Okumu et al. [21]. Such cumulative impacts were also demonstrated in a trial of odour-baited trap in western Kenya, which achieved significant reductions in densities of *An. funestus* mosquitoes, and subsequently reduced malaria incidence by 40.8% and prevalence by 28.9% [46]. The potential benefits of traps, used alone or in combination with transfluthrin-treated eave ribbon, could however not be detected at the scale of this current study.

Most *Anopheles* traps have been developed for surveillance, and their applications as mass-trapping interventions remain circumstantial, often dependent on the type of lure used and the density of traps relative to human and animal hosts. In this study, Mosclean trap was selected and used as an intervention based on a previous study [37], where the trap was demonstrated to catch more mosquitoes than other commonly used traps such as CDC-light traps, BG-sentinel, and Suna traps.

One limitation of this study is that it was conducted in semi-field settings under controlled environment and using laboratory-reared mosquitoes. Second, the sentinel hut remained the same throughout the tests, and therefore lacked the natural variability associated with actual field settings. Lastly, the study used untreated mosquito nets for primary protection in all the huts instead of insecticide-treated bed nets (ITNs). The method for assessing exposure also did not consider the exposure to bites that occur when people are indoors but not yet under their nets. Instead the method used assumed that people went to bed as soon as they were indoors. Field validation of the findings may therefore be necessary, in different geographical locations, with different vector species composition. Preferably such studies should be in areas with pyrethroid resistant mosquitoes so that both the mortality and repellency effect, and any other effects of the intervention can be validated. An alternative validation strategy could be mathematical simulations, such as previously done for odour-baited traps [21]. Future research is needed to establish public health impact and inform successful execution of the technology.

## Conclusion

Eave ribbons treated with protect both users and non-users against malaria mosquitoes. The benefits to the non-users is highest where coverage of eave ribbons exceeds 80%. Besides, most of the gains accrued by the non-users was in form of outdoor protection, even though percentage overall protection for both users and non-users was higher indoors. The mosquito-killing property of transfluthrin can magnify the communal benefits by limiting unwanted diversion to non-users, but should be validated in field trials against pyrethroid-resistant vectors. The potential of the UV–LED traps as an intervention alone or alongside eave ribbons was however undetectable at the scale of this study. Overall, these findings extend the evidence that transfluthrin-treated eave ribbons could potentially complement ITNs. This study highlights the potential for eave ribbons to protect people in peri-domestic space before sleeping hours when ITN use is not feasible.

## Data Availability

The dataset for this study is available from the corresponding author upon request.

## Abbreviations

ITNs: insecticide-treated bed nets
UV–LED: ultraviolet-light emitting diodes
CO_2_: carbon dioxide
HLC: human landing catch
